# Study of Fingerprint Patterns in Population of a Community

**DOI:** 10.31729/jnma.4621

**Published:** 2019-10-31

**Authors:** Iju Shrestha, Banshi Krishna Malla

**Affiliations:** 1 Department of Anatomy, Kathmandu Medical College and Teaching Hospital, Duwakot, Bhaktapur

**Keywords:** *fingerprint pattern*, *gender identity*, *sexual dimorphism*

## Abstract

**Introduction:**

Fingerprints, serve as one of the crucial tools for identification of the individual for various purposes. Sex, being one of those tools, researchers have suggested the use of fingerprints for gender identification. The objective of the study was to observe the distribution of various fingerprints patterns in the population of a community, together with the most prevalent pattern.

**Methods:**

This descriptive cross-sectional study was conducted in the population of Duwakot VDC, Bhaktapur from May 2019 to July 2019. Ethical clearance was obtained from the Institutional Review Committee with reference no. 2812201804. One hundred and ninety-six individuals of 18 to 60 years of age were enrolled. Fingerprints of all ten fingers were taken and studied to see the distribution pattern and analyzed for gender differences. Simple random sampling was done and the sample size was calculated with a prevalence of 50%. The data obtained were computed and analyzed using Excel to find the results.

**Results:**

The study showed the highest frequency of loops 1033 (52.71%) followed by whorls 537 (27.38%), arches 537 (27.38%) and composite pattern 300 (15.28%). The radial loops were observed more in the males 397 (5.54%) of total males whereas ulnar loops were observed more in the females 636 (96.38%) of total females. Among whorls, the concentric whorls were seen more in males 245 (52.03%) whereas the spiral whorls were seen more in the females 292 (53.27%).

**Conclusions:**

For standard authenticity of the sexual dimorphism, fingerprint patterns, can also be considered for gender identification purposes.

## INTRODUCTION

Fingerprints serve as one of the crucial pieces of evidence found at the scene of occurence, giving a positive means of identification.^1^ The fingerprints are taken as the most reliable criteria for identification, as they are constant and individualistic.^2^

Due to the uniqueness of fingerprints, differentiation of even identical twins can be done with ease, where DNA profiling proves futile.^1^ Sex is among the most important information that discriminates individuals; researchers have suggested the use of fingerprint for gender identification which can be more helpful in shortlisting the suspects.^3^ However, relatively less research has been done for its use in this field of gender identification.

The present study was done to study various patterns of fingerprints and their distribution in the population of Duwakot, Bhaktapur, together with the most prevalent fingerprint pattern and to establish the relationship between the fingerprints and gender of an individual.

## METHODS

This descriptive cross-sectional study was carried out in the population of Duwakot VDC, Bhaktapur from May 2019 to July 2019. The study enrolled 196 individuals of eighteen to sixty years of age. Ethical clearance from the Institutional Review Committee –Reference No. 2812201804 was obtained. The informed consent of the participants was taken prior to the procedure. Age, sex and the fingerprints of all ten fingers of the participants were recorded. Subjects with a major deformity (congenital/accidental) on the upper extremity (syndactyly, polydactyly) or with leprosy and with gender identity disorder were not included in the study. Fingerprints, as they are considered as a sensitive matter, subjects who did not give consent were also not included.

For the fingerprints, the participant was asked to stand in front and at a forearm length from the paper on which fingerprint was to be taken. The ink was applied on the fingers from the stamp pad in a proximo-distal direction. The print was then taken by placing the finger at a right angle to the surface of the paper. The finger was then pressed lightly on the paper and rolled uniformly in radio-ulnar direction. Prints taken were further scanned and enlarged for the study.^1^

Simple random sampling was done and the sample size was calculated with prevalence 50%.

The sample size (n) was calculated as follows:

n = Z^2^ × p × q/e^2^

   = (1.96)^2^ × 0.5 × 0.5/(0.7)^2^

   = 196

Where,

Z= 1.96 for 95% confidence interval
p = 0.5
q = 1-p
e= margin of error= 7%
N = population of Duwakot according to census 2011, Central Bureau of Statistics, Nepal

For finite population,

N_0_ = n/1 +(n/N)

     = 196/1+(196/10,461)

     = 196/(1+0.0187)

     = 196/1.0187

     = 193

Hence, the total sample size taken was 196.

The data obtained were computed and analyzed using Excel to tabulate the results.

## RESULTS

**Figure 1:**
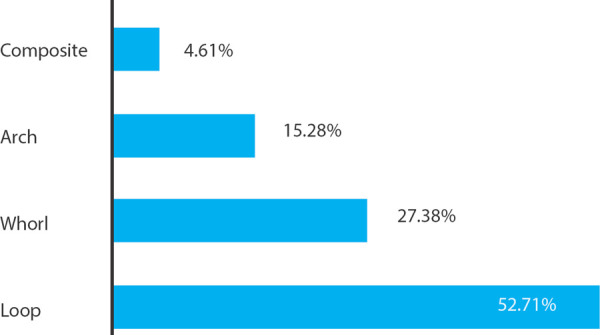
Distribution of the fingerprint patterns in total subjects.

In this study, a hundred and ninety-six subjects were included making 1960 total fingerprints. Among the fingerprints recorded, the maximum number was of loops 1033 (52.71%) followed by whorls 537 (27.38%) and arches 300 (15.28%). The last occurrence was seen in the composite pattern 90 (4.61%) (Figure 1).

**Figure 2:**
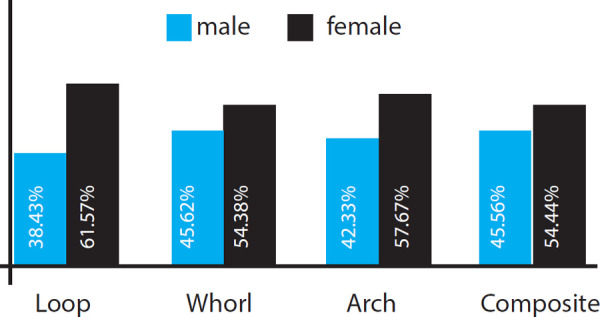
Distribution of the fingerprint patterns in males and females.

The frequency of the patterns was studied for males and females. Loop was seen in 397 males and 636 females whereas the whorls in 245 males, 292 females. Similarly, 127 males, 173 females and 41 males, 49 females were seen with the occurrence of arches and composites respectively. In the overall distribution, all four patterns were seen to occur more in females (Figure 2).

The patterns of the loop (total of males 397 and females 636) and whorl (male 245 and female 292) were further analyzed and divided into radial and ulnar loops and concentric and spiral whorls respectively.

**Figure 3:**
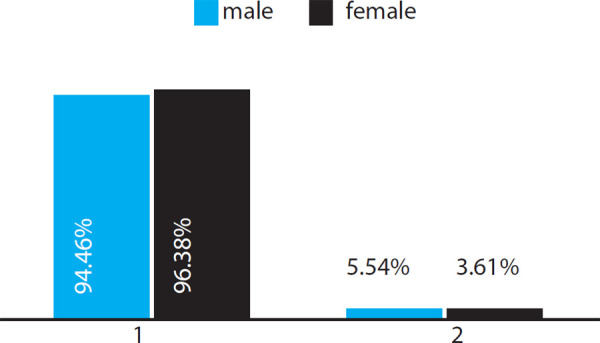
Distribution pattern of the loop in males and females.

Observation in the total male and female subjects respectively showed that the ulnar loops (1) were observed more in the females (613 out of 636) whereas radial loops (2) were observed more in the males (5.54%, 22 out of 397 males) in comparison to females (3.61%, 23 out of 636 females) (Figure 3).

**Figure 4:**
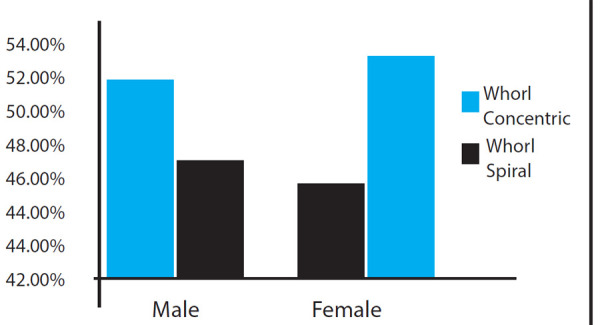
Distribution pattern of whorl in males and females.

Among the whorls observed, the concentric whorls were seen more in males (128 out of 245 males) whereas the spiral whorls were seen to occur more in the females (156 out of 292 females) (Figure 4).

## DISCUSSION

Fingerprint patterns, due to their uniqueness, play an important role in the identification and verification of an individual. The distribution of the fingerprint patterns can vary in different communities, still maintaining the individuality.

In this study, a total of 1960 fingerprints were taken which revealed the loops as the most common pattern followed by the whorls and the arches. This finding was consistent with the studies done by Karki and Singh and Shukla et al. where they have reported higher frequencies of loops followed by whorls and arches.^3,4^ Our study also matched the study conducted in Nepalese citizens by Shrestha et al, where loops were seen to occur in about 52.90% of the total and the whorls were 30.00%. Though slight differences occurred in the patterns of arches and composites where it was reported 10.8% and 6.1% respectively.^5^ A study done in the Sinhalese population in Srilanka has also reported 59.72% loops and 35.53% whorls which were similar to our study.^6^ However, the frequency of the arches reported in their study showed only 4.75% of arches whereas in our study it was a little higher of 15.28%.

The least frequency, as shown by our study was of the composite patterns (4.61%). Other studies done have also shown the composites to be the least frequent.^5,7^ In the same way, Nithin et al. reported the arches as their least occurring fingerprint in the South Indian population with 6.2%.^8^ Similarly, Qayyum et al. have also reported the least frequency of arches in the population of Rawalpindi with 8.4%.^9^

In observation of the fingerprint pattern distribution in our study, all four patterns were found to be in higher frequencies in females. Some studies have reported the higher occurrence of loops in males and whorls in females.^5,7^ Other studies, in contrast, have shown the loops to be more common in females and the whorls in the males.^3,6,8,10^

Looking into the subtypes of loops and whorls, the ulnar loop was slightly more common in females (96.38%) than in males (94.46%), whereas the radial loop was more common in the males (5.54%). On the contrary, Reddy et al have reported a higher frequency of radial loop in females.^11^ Amongst the whorls, concentric whorl occurred more in males (52.03%) and the spiral whorl occurred more in females (53.27%). A similar finding of higher occurrence of the spiral whorl in females has been reported by Nithin et al.^8^

The occurrence of the fingerprint patterns in general as shown by our study and other different studies were almost similar with few differences. However, differences in occurrence of the patterns in the sexes were observed. Hence, sexual dimorphism of the fingerprint patterns may be attributed to differences in heritability and developmental variation among sexes.

The limitation of the study may be taken as a lesser number of male participants since the data of this study was taken during the daytime when most of the males were out of the house for their work.

## CONCLUSIONS

Fingerprints, undoubtedly serve as one of the crucial tools for the identification of the individual for various purposes. Owing to the presence of differences in the patterns in the sexes, it can also be considered in gender identification purposes. However, more detailed studies have to be conducted for the standard authenticity of sexual dimorphism.

## References

[1] Mandrah K, Kanwal NK (2016). A Preliminary Study on Assertion of Hand from Whorl Pattern on Thumb.. J Med toxicol clin forensic med..

[2] Kanchan T, Chattopadhyay S (2006). Distribution of Fingerprint Patterns among Medical Students.. J Indian Acad Forensic Med..

[3] Karki RK, Singh PK (2014). Gender determination from fingerprints. JUCMS..

[4] Shukla S, Sharma N, Jain SK, Budhiraja V, Rastogi R, Garg R, et al (2016). A Study of Sexual Dimorphism in Finger Print Pattern in Indian Population. Ann Int Med Den Res..

[5] Shrestha DB, Gupta VP, Chaurasiya PS, Shrestha S, Chaudhary S, Aryal L (2016). Study of Correlation between Different Fingerprint Patterns, Blood Groups, and Social Behavior among Medical Students Nepalese Citizens).. Pac J Sci Technol..

[6] Wijerathne BTB, Rathnayake GK, Adikari SC, Amarasinghe S, Abhayarathna PL, Jayasena AS (2013). Sexual dimorphism in digital dermatoglyphic traits among Sinhalese people in Sri Lanka. J Physiol Anthropol..

[7] Narayana BL, Rangaiah YKC, Khalid MA (2016). Study of fingerprint patterns in relation to gender and blood group.. J Evolution Med Dent Sci..

[8] Nithin MS, Rema P, Venugopalan Nair B. (2015). Study of Fingerprint Patterns in South Indian Population.. J Indian Acad Forensic Med..

[9] Qayyum R, Mateen A, Hameed S (2013). Pattern of Finger Prints in The Population of Rawalpindi. JRMC..

[10] Rastogi P, Pillai KR (2010). A Study of Fingerprints in Relation to Gender and Blood group. J Indian Acad Forensic Med..

[11] Reddy M, Karumanchi S, Anasuya K (2011). A Study of Finger Prints: Bilateral Asymmetry and Sex Difference in the Region of Andhra Pradesh.. J Clin Diagn Res..

